# Quantification of transmission of foot-and-mouth disease virus caused by an environment contaminated with secretions and excretions from infected calves

**DOI:** 10.1186/s13567-015-0156-5

**Published:** 2015-04-17

**Authors:** Carla Bravo de Rueda, Mart CM de Jong, Phaedra L Eblé, Aldo Dekker

**Affiliations:** Central Veterinary Institute (CVI), part of Wageningen UR, P.O. Box 65, 8200 AB Lelystad, The Netherlands; Department Quantitative Veterinary Epidemiology, Wageningen University, P.O. Box 338, 6700 AH Wageningen, The Netherlands

## Abstract

Foot-and-mouth disease virus (FMDV) infected animals can contaminate the environment with their secretions and excretions. To quantify the contribution of a contaminated environment to the transmission of FMDV, this study used calves that were not vaccinated and calves that were vaccinated 1 week prior to inoculation with the virus in direct and indirect contact experiments. In direct contact experiments, contact calves were exposed to inoculated calves in the same room. In indirect contact experiments, contact calves were housed in rooms that previously had held inoculated calves for three days (either from 0 to 3 or from 3 to 6 days post inoculation). Secretions and excretions from all calves were tested for the presence of FMDV by virus isolation; the results were used to quantify FMDV transmission. This was done using a generalized linear model based on a 2 route (2R, i.e. direct contact and environment) SIR model that included information on FMDV survival in the environment. The study shows that roughly 44% of transmission occurs via the environment, as indicated by the reproduction ratio ${{\widehat{R}}_0}^{2R} environment$ that equalled 2.0, whereas the sum of ${{\widehat{R}}_0}^{2R} contact$ and ${{\widehat{R}}_0}^{2R} environment$ equalled 4.6. Because vaccination 1 week prior to inoculation of the calves conferred protective immunity against FMDV infection, no transmission rate parameters could be estimated from the experiments with vaccinated calves. We conclude that a contaminated environment contributes considerably to the transmission of FMDV therefore that hygiene measures can play a crucial role in FMD control.

**Electronic supplementary material:**

The online version of this article (doi:10.1186/s13567-015-0156-5) contains supplementary material, which is available to authorized users.

## Introduction

Foot-and-mouth disease virus (FMDV) is the causative agent of foot-and-mouth disease (FMD), a highly contagious disease of livestock. Outbreaks of FMD cause vast sums of money to be spent, to reduce its incidence to low levels [1]. Control measures to restrict the spread of FMDV include movement restrictions, but even when movement restrictions are applied, these do not always prevent new outbreaks (for example in the 2001 FMD epidemic in United Kingdom [2]). Since these restrictions mean that livestock are not allowed to move between farms, direct contact cannot be the (major) cause of transmission, so other, indirect, routes must play a role.

Because most of the secretions and excretions of FMDV infected animals contain virus [3], environmental contamination with secretions and excretions containing FMDV was considered to be one of the causes of FMDV spread [4]. This conclusion was supported by the fact that FMDV remains in the environment, for at least 24 h, after infected animals are killed [5]. Moreover, as studies on survival of FMDV in secretions and excretions have shown, detectable amounts of FMDV persist in the environment (for example, in manure) for up to 14 weeks due to the thermal stability of the virus [6,7]. The suspicion that an environment contaminated with secretions and excretions from FMDV infected animals contributes to the transmission of FMDV has likewise persisted.

SIR (susceptible-infected-recovered) models have been used to model the role of the environment in the transmission of different pathogens [8-12]. Although transmission of FMDV has been quantified in animal experiments [13,14] using a stochastic SIR model [15] and a transient-state algorithm [16], such studies have neither modelled nor quantified the contribution of the environment. In addition, FMDV transmission is known to be reduced through vaccination [17], and that vaccinating 2 weeks before inoculation with the virus reduces the reproduction ratio *R*_0_ to a value below 1 [18]. However, it is unknown whether this could be accomplished through earlier vaccination.

Thus, the aim of the present study is twofold: to utilize a 2 route-SIR model i.e. with both direct contact and indirect (environment) routes, to quantify the contribution of a contaminated environment to the transmission of FMDV, and to examine whether vaccination one week before inoculation with the virus could reduce FMDV transmission through either direct contact or via the environment. As this article shows, a contaminated environment contributes considerably to the transmission of FMDV, and vaccination of cattle 1 week prior to inoculation with the virus does confer protective immunity against FMDV infection.

## Materials and methods

### Experimental design

We used 46 female calves, aged between 6 and 7 months, born and raised in The Netherlands on conventional dairy farms. Our experiments were performed in rooms approximately 10 m^2^ inside the biosecurity facilities of the Central Veterinary Institute (CVI, Lelystad, The Netherlands). The settings for temperature and humidity in the stables were 20 – 24 °C and 40 – 70% relative humidity respectively. The experiments received ethical approval from the animal experiment committee of the CVI in accordance with Dutch law. The experiments with non-vaccinated calves and the experiments with vaccinated calves were performed sequentially. During the experiments, all calves were inspected daily for clinical signs of FMD. In these inspections, rectal temperature above 39.5 °C was considered fever [19] and the calves were checked for the presence of FMD lesions i.e. vesicles. During inspection and/or sampling, animal caretakers changed coveralls and gloves between animal rooms. The animal rooms in which the indirect transmission experiments were performed were not cleaned with water; instead, animal waste was swept daily with a broom to the drainage.

### Challenge virus and vaccine

Virus inoculation was performed intranasally using FMDV Asia-1 TUR/11/2000. The inoculum contained 10^6.1^ plaque forming units (pfu)/mL (titrated on primary lamb kidney cells). Each inoculated calf received 1.5 mL of inoculum per nostril. The vaccine used was a freshly prepared inactivated FMDV Asia-1 Shamir vaccine, prepared in a double water-in-oil emulsion. The potency of a similarly prepared vaccine was previously determined at > 6 PD_50_ (at 28 days post vaccination).

### Direct contact experiments

In both vaccinated and unvaccinated scenarios, 10 calves were randomly assigned to 5 animal rooms in pairs i.e. 2 calves per room. On the day of inoculation i.e. 0 days post inoculation (dpi), 1 calf from each pair was moved to a separate animal room and inoculated with FMDV. Eight hours after inoculation, these calves were reunited with their original roommates. In the experiment in which vaccinated calves were used, all 10 calves were vaccinated intramuscularly with 2 mL of vaccine one week before inoculation (−7 dpi). The direct contact experiments ended at 14 dpi, assuming this duration could allow transmission to occur.

### Indirect contact experiments

**Figure 1:**
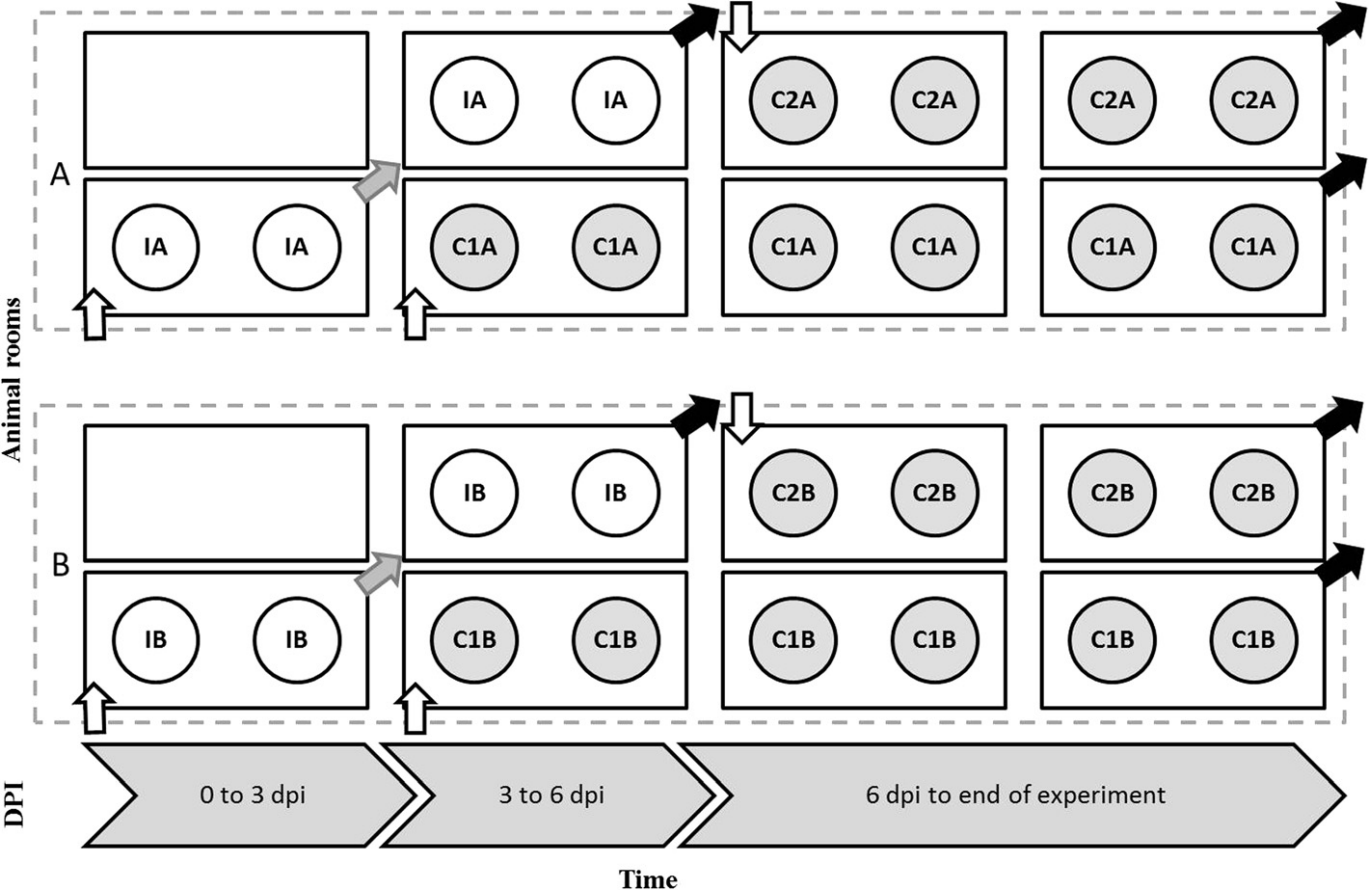
**Indirect contact experiment design.** Panels **A** and **B** represent groups A and B. IA and IB, calves inoculated at 0 days post infection (dpi); C1A and C1B, contact exposed calves to contaminated environment from 0 to 3 dpi; C2A and C2B, contact exposed calves to contaminated environment from 3 to 6 dpi. Grey arrows indicate movement of animals to an (− other) animal room. Black arrows indicate movement of animals for euthanasia.

This experimental design is shown in Figure 1. In both vaccinated and unvaccinated scenarios, 4 calves were inoculated with FMDV at 0 dpi (2 pairs (groups A and B) of inoculated calves, IA and IB). Eight hours after inoculation, they were moved into 2 animal rooms to which they had been randomly assigned, 2 calves per room. At 3 dpi, the inoculated calves were moved to 2 new animal rooms. Subsequently, 1 pair of non-vaccinated contact calves (contacts 1, C1A and C1B) was moved into each of the animal rooms that had been contaminated by the inoculated calves. The inoculated calves stayed in their new rooms from 3 to 6 dpi; at 6 dpi, they were removed from the animal rooms and euthanized. On the same day, each of these now-contaminated rooms was allocated to a pair of non-vaccinated contact calves (contacts 2, C2A and C2B). In the experiment in which vaccinated calves were used, at −7 dpi the 4 inoculated calves were vaccinated intramuscularly with 2 mL of vaccine. The 8 contact calves were not vaccinated. The indirect contact experiments ended at 20 dpi.

### Vaccine controls

During the experiment with vaccinated calves, 2 additional calves were vaccinated and used as vaccine control group to evaluate the serological response of the calves in the absence of infection; these controls were housed together in a separate animal room.

### Sampling

Oropharyngeal fluid (OPF) swabs, heparinised blood, urine and faeces samples were collected daily from each calf from 0 dpi until the end of the experiment. OPF was collected by inserting a cotton gauze with a 25 cm long forceps into the mouth of the calves and by rubbing the surface of the oropharyngeal cavity. In the laboratory, the pieces of cotton gauze were immersed in 4 mL of Eagle’s minimum essential medium (EMEM) containing 2% fetal calf serum (FCS) and 10% antibiotics solution (ABII: 1000 U/mL of penicillin, 1 mg/mL of streptomycin, 20 μg/mL of amphotericin B, 500 μg/mL of polymixin B, and 10 mg/mL of kanamycin). After 20 min of incubation at environmental temperature, the samples were centrifuged (2500 rpm for 15 min). Samples were stored at −70 ***°***C until virus isolation and real-time reverse transcriptase polymerase chain reaction (RT-PCR) analysis.

Heparinised blood samples (10 mL per calf) for virus isolation were taken daily, while clotted blood samples (10 mL per calf) for serology were taken twice per week. Blood samples were centrifuged at 2500 rpm for 15 min. Plasma was stored at −70 ***°***C until virus isolation analysis and serum was stored at −20 ***°***C until serological analysis. Urine samples were collected, as calves were stimulated to urinate spontaneously by rubbing the skin next to the vulva. Urine samples were collected into sterile plastic containers. In the laboratory, 800 μL of urine was mixed with 200 μL of a 50% FCS, 50% ABII solution and stored at −70 ***°***C until virus isolation analysis.

Faeces samples were collected from the rectum. In the laboratory, the faeces was suspended 1:10 (w/v) in EMEM containing 10% FCS and 10% ABII solution, and vortexed with glass beads. After 20 min of incubation at environmental temperature, the suspension was vortexed and centrifuged (3000 rpm for 15 min). The supernatants were stored at −70 ***°***C until virus isolation analysis.

### Virus detection

All OPF, heparinised blood, urine and faeces suspension samples were tested for presence of FMDV by plaque count on monolayers of secondary lamb kidney cells (virus isolation, VI). Samples were tested in 2 wells of a six-well plate using 200 μL per well, as previously described [20]. All OPF samples were also tested for presence of FMDV by RT-PCR. RNA isolation was performed using the Magna Pure LC total Nucleid Acid Isolation kit® (Roche) and the MagNa Pure 96 system® (Roche). Isolated RNA was tested in a LightCycler 480 Real-Time PCR System® (Roche) using a QuantiFast Probe RT-PCR kit® (Qiagen), all in accordance with the manufacturers’ instructions. The primers, probes and test protocol used have been previously described [21].

### Statistical analysis of virus secretions and excretions

Using data from both the direct and the indirect contact experiments, we calculated, for individual animals, the area under the curve (AUC) of the virus titres. The AUC represents the total amount of FMDV that was secreted and excreted by the infected calves during the experiment. The AUCs were calculated for each calf using the non-logarithm-transformed virus titres observed in its OPF swabs, urine and faeces samples. In the statistical analysis, the logarithm of the AUC was used (log AUC). The maximum FMDV log titres found in OPF swabs, urine and faeces samples from each calf were also calculated. The duration (in days) of FMDV secretion and excretion in OPF swabs, urine and faeces samples was calculated for each calf, counting from the first day until the last day the calf tested positive in the virus isolation assay (in either OPF swabs, urine or faeces samples). A Kruskal Wallis test was used to test whether differences existed between the experimental groups (i.e. inoculated calves, direct contacts, indirect contacts C1 and indirect contacts C2) for either the log AUC, the maximum FMDV log titres or the duration of FMDV secretion and excretion. The log AUC and the maximum FMDV log titres were tested for each type of sample (OPF swabs, urine and faeces). The duration of FMDV secretion and excretion was tested using data from OPF swabs, urine and faeces samples combined.

### Antibody detection

A commercially available ELISA (PrioCHECK® FMDV NS, Prionics) was used to detect antibodies against non-structural proteins of FMDV. The test was performed in accordance to the manufacturer’s instructions. This test detects antibodies against the non-structural protein 3B of FMDV and differentiates infected from non-infected animals in both non-vaccinated and vaccinated animals. Samples were considered to be positive when the percentage of inhibition was ≥ 50%. The virus neutralization test (VNT) was performed as previously described [22] but using BHK-21 cells instead of porcine kidney cells. Titres were determined against both the vaccine strain (Asia-1 Shamir) and the challenge strain (Asia-1 TUR/11/2000). Samples were considered to be positive when the titres were above 1.2 ^10^log (cut-off of validated diagnostic test) using the Asia-1 Shamir strain and 0.6 ^10^log (cut-off based on the score of control samples) using the Asia-1 TUR/11/2000 strain.

### Quantification of the FMDV survival rate

The FMDV survival rate (σ day^−1^), needed for the calculation of the contribution of the environment (E_t_) to the transmission of FMDV, was calculated using published data on FMDV thermal inactivation combined with own laboratory data. Because the temperature in the animal rooms was approximately 20 °C during the experiments, the survival rate σ was estimated at 20 °C. The lowest, middle and highest estimates of the time needed for a 10-fold reduction in FMDV titres at 20 °C was used to calculate the FMDV survival rate σ. An additional file shows the calculation of the FMDV survival rate σ in more detail (Additional file 1 with references [7,20,23-27]).

### Quantification of FMDV transmission

#### Transmission rate parameters: β, β_contact_ and β_environment_

**Figure 2:**
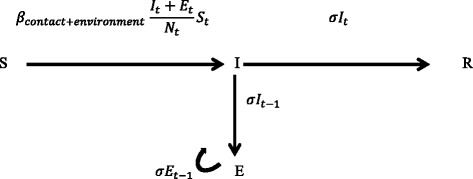
**The 2R- SIR model.** The combined transmission rate parameter (*β*_*contact+environment*_) depends on the number of infectious calves (I_t_) and/or on the amount of virus in the environment (E_t_). E_t_ depends on FMDV secretion and excretion by the infected calves on previous days (t-1) and on the remaining amount of FMDV in the environment weighted by σ.

The transmission rate parameter β is defined as the average number of new infections caused by one typical infectious individual per day in a totally “susceptible” (not infected) population [16,28] (Additional file 2: equations 1 and 2, with references [16,28,29]). For the analysis, as described previously [28], it was assumed that the calves were infectious (I) when one of their samples (OPF swabs, urine or faeces) tested positive in the virus isolation assay at the start of the time interval. Contact animals were considered cases (C) when one of their samples (OPF swabs, urine or faeces) tested positive, for the first time, in the virus isolation assay at the end of the time interval. The number of new cases (C) during that time interval is binomially distributed with probability *p* (which is a function of the transmission rate parameter β, the number of infected animals (I_t_) and the total number of animals (N)) and with binomial total S_t_, the number of susceptible animals. Thus, the probability of a single susceptible animal becoming infected during a period Δt is, $p=1-{e}^{-{e}^{C_0}\times \frac{I_t}{N_t}\times \Delta t}$, where ${e}^{C_0}$ is the transmission rate parameter β. To quantify β, the data from the direct contact experiment were analysed using a generalized linear model (GLM) [30]. The GLM is based on the binomial distribution and the above-mentioned expression for *p*, using a complementary log-log link function, S as binomial total, a binomial error function and with $\log \left(\frac{I_t}{N_t}\times \Delta t\right)$ as offset [16,28]. This model will be hereinafter referred to as the 1 route-SIR (1R-SIR) model. To quantify the contribution of the environment to the transmission of FMDV, as an extra route to the 1R-SIR model (Figure 2), we included the environment (E). In the new 2 route-SIR model (2R-SIR) we additionally assumed that the amount of FMDV present in the environment on a specific day (E_t_) depends on the secretion and excretion of FMDV by infectious individuals (either I or C) on the previous days, as well as on the remaining FMDV in the environment (E_(t-1)_), both weighted (discounted) by the FMDV survival rate (σ). E_t_ is represented by the following equation: *E*_*t*_ = *σI*_(*t* − 1)_ + *σC*_(*t* − 1)→ *t*_ + *σE*_(*t* − 1)_ with starting condition E_0_ = 0 (Additional file 2: equation 3). We performed a sensitivity analysis in which we multiplied the new cases (C) in the equation above either by 0 or by 0.5, instead of 1 as it is in the above equation for E_t_, to check whether this affected the outcome. Additionally, we performed a sensitivity analysis in which we considered a latent period (counting the inoculated calves as infected but not yet infectious, (1, 2 and 3 days before virus shedding was detected), to check whether the use of an SEIR (susceptible-exposed-infected-recovered) instead of an SIR model would lead to different results for the estimated β and R values (i.e. if β is underestimated) and whether this affected the estimation of the environmental component.

In the 2R-SIR model, there are 2 ways by which the susceptible calves (S_t_) can become infected: (1) because they have been in direct contact with an infectious calf (I_t_) i.e. being in the same room at the same day as an infectious calf and/or (2) because they have been in contact with a contaminated environment (E_t_) i.e. being in an animal room that housed previously one or more infectious individuals (Figure 2). By using the 2R-SIR model, we quantified the transmission rate parameters *β*_contact_ and *β*_environment_. As in the definition of β the transmission rate parameter *β*_contact_ is defined as the average number of new infections per day caused by direct contact to one typical infectious individual in a fully susceptible population. The transmission rate parameter *β*_environment_ is defined as the average number of new infections per day caused by virus in the environment, where the unit of infectivity is equal to the amount of virus secreted and excreted during one day by an infectious animal. An additional file shows the 2R-SIR model in more detail (Additional file 2: equations 4 to 6). In the 2R-SIR model, the number of new cases (*C*_*t*→ (*t* + 1)_), whether caused by I_t_ and/or E_t_, is binomially distributed with parameter *p* as before (see also below) but now $\beta ={e}^{C_0+{C}_1\times fe}$ where ${\mathrm{f}}_{\mathrm{e}}=\frac{E_t}{I_t+{E}_t}$ is the fraction of transmission by the environment and therefore its regression coefficient measures the extra infectivity contributed by the environment. When only direct contact can occur, f_e_ is 0 and thus ${\beta}_{contact}={e}^{C_0}$. When only environmental exposure can occur, f_e_ is 1 and ${\beta}_{environment}={e}^{C_0+{C}_1}$ (Additional file 2). The latter expression contains c_0_ + c_1_ and thus c_1_ is the extra transmission for each unit of infectivity through the environment as compared to one unit through direct contact. Thus the probability of a susceptible animal becoming infected during a period Δt is $p=1-{e}^{-{e}^{C_0+{f}_e\times {C}_1}\times \frac{I_t+{E}_t}{N_t}\times \Delta t}$ (Additional file 2: equation 6). To quantify β_contact_ and β_environment_ we analysed the combined data from both the direct contact experiment and the indirect contact experiment using a GLM. The GLM was based on the binomial distribution and the above mentioned expression for *p* using a complementary log-log link function, S as binomial total, a binomial error function, f_e_ as the explanatory variable [28] and with $\log \left(\frac{I_t+{E}_t}{N_t}\times \Delta t\right)$ as offset (Additional file 2: equations 7 and 8). To test whether *β*_contact_ and *β*_environment_ were significantly different from each other, we used the Wald test on the regression coefficient of f_e_. Both analyses (of the 1R-SIR and of the 2R-SIR models) were performed using the statistical program R [31] and the package stats.

#### Infectious periods: T and τ

The infectious period T was defined as the average infectious period of the inoculated calves that caused transmission from the direct contact experiment. The infectious period of each inoculated calf was defined as the time between the first and the last day on which FMDV was detected (by virus isolation) in OPF swabs, urine, or faeces samples. The 95% confidence intervals (CI) of $\widehat{\mathrm{T}}$ were calculated using the logarithm of T (log T) and the variance of log T i.e. ${e}^{logT\pm 1.96\sqrt{var\kern0.5em (logT)}}$. The infectious period τ represents the infectious period of the contaminated environment. The calculation of τ was based on the amount of infectious material present in the environment (E_t_, used in the 2R-SIR model). Considering the loss of infectiousness due to inactivation at environmental temperature, τ was calculated by taking the sum of geometric series: $\tau ={\displaystyle {\sum}_{i=1}^{\infty }{\sigma}^i\widehat{T}}=\widehat{T}\left(\frac{1}{1-\sigma }-1\right)$ where σ is the survival rate of FMDV and $\widehat{T}$ is the estimated average infectious period of the inoculated calves in the direct contact experiment. The method allowed us to obtain an average period over which one infectious animal contributes to the contamination of the environment, weighted for the amount of infectious material relative to the amount secreted and excreted by an infectious animal on one day. The 95% CI of $\widehat{\tau}$ was calculated using the 95% CI of $\widehat{T}$.

### Reproduction ratio *R*_0_

#### Using the 1R-SIR model: R_0_^1R^

The reproduction ratio *R*_0_^*1R*^ is defined as the average number of new infections caused by one typical infectious individual in a population made up entirely of susceptible individuals [32]. *R*_0_^*1R*^ was estimated by multiplying the transmission rate parameter $\widehat{\beta}$ by the infectious period $\widehat{T}$. The 95% CI of ${{\widehat{R}}_0}^{1R}$ was calculated using the variance and the regression constant of the GLM result (log β) and the variance and the logarithm of the average infectious period T, i.e. ${e}^{log\beta + logT\pm 1.96\sqrt{var\kern0.5em \left( log\beta \right)+var\kern0.5em \left( log T\right)}}$.

#### Using the 2R-SIR model: R_0_^2R^_contact_, R_0_^2R^_environment_ and R_0_^2R^

The reproduction ratio *R*_*0*_^*2R*^ is defined as the average number of new infections caused by both direct contact to one typical infectious individual in a population made up entirely of susceptible individuals and the virus left in the environment by that one typical infectious individual on the previous days. Both *R*_*0*_^*2R*^_*contact*_ and *R*_*0*_^*2R*^_*environment*_ were estimated using the results from the 2R-SIR model, i.e. estimated transmission rate parameter $\widehat{\beta}$_contact_ and $\widehat{\beta}$_environment_. The *R*_*0*_^*2R*^_*contact*_ was estimated by multiplying ${\widehat{\beta}}_{\mathrm{contact}}$ by the infectious period $\widehat{T}$. The *R*_*0*_^*2R*^_*environment*_ was estimated by multiplying ${\widehat{\beta}}_{\mathrm{environment}}$ by the infectious period $\widehat{\tau}$. Subsequently *R*_*0*_^*2R*^ was estimated by summing ${{\widehat{R}}_0}^{2R} contact$ and ${{\widehat{R}}_0}^{2R} environment$. The ${{\widehat{R}}_0}^{2R} contact$ is the contribution to ${{\widehat{R}}_0}^{2R}$ by direct contact to virus from an infectious individual (on the day virus secretion and excretion is detected by virus isolation). The ${{\widehat{R}}_0}^{2R} environment$ is the contribution to ${{\widehat{R}}_0}^{2R}$ by the virus left in the environment by infectious individuals on previous days. The 95% CI of ${{\widehat{R}}_0}^{2R} contact,\kern0.5em {{\widehat{R}}_0}^{2R} environment$ and of ${{\widehat{R}}_0}^{2R}$ were calculated. For this purpose, we used the variances and the regression constants (see above c_0_ and c_1_ in equation for *p*) of the GLM results (log *β*_*contact*_ or log *β*_*environment*_) and the variances and the logarithm of the average infectious periods (log T or log τ). Thus, the 95% CI of the ${{\widehat{R}}_0}^{2R} contact$ is ${e}^{log{\beta}_{contact}+ \log T\pm 1.96\sqrt{\operatorname{var}\kern0.5em \left( log{\beta}_{contact}\right)+var\left( log T\right)}}$ and, the 95% CI of the ${{\widehat{R}}_0}^{2R} environment$ is ${e}^{log{\beta}_{environment}+ \log T+ loga\pm 1.96\sqrt{\operatorname{var}\kern0.5em \left( log{\beta}_{environment}\right)+var\left( log T\right)}}$ where a is $\left(\frac{1}{1-\sigma }-1\right)$. As ${{\widehat{R}}_0}^{2R}$ is the sum of ${{\widehat{R}}_0}^{2R} contact$ and ${{\widehat{R}}_0}^{2R} environment$, its variance is $var\left({e}^{\log \left({\widehat{R}}_0^{2R}\right)}\right)=var\left({e}^{\log \left({\widehat{R}}_0^{2R} contact\right)}\right)+var\left({e^{\log}}^{\left({\widehat{R}}_0^{2R} environment\right)}\right)$ and although this is not a linear function we calculated the 95% CI of the ${{\widehat{R}}_0}^{2R}$ using: $var\left({e}^{\log \left({\widehat{R}}_0^{2R}\right)}\right)={e}^{\operatorname{var}\left( log\left({\widehat{R}}_0^{2R} contact\right)\right)}+{e}^{var\left( log\left({\widehat{R}}_0^{2R} environment\right)\right)}$.

#### Using the final size model: R_0_^FS^

The transmission parameter *R*_*0*_ can also be estimated based only on the final outcome (the final size of the experiment, FS) [33]. We estimated the *R*_*0*_^*FS*^ based on the total number of infected calves at the end of the direct contact experiment under the assumption that the epidemic process ended before the experiment stopped [34]. The animals were considered infected when one or more of their samples tested positive in the virus isolation assay. Because in the direct contact experiment we got all 4 contacts infected in the 4 pairs in which the inoculated calf was considered to be infectious, we used continuity correction, i.e. 3.5 infections in 4 experiments, to avoid an infinite estimate for *R*_*0*_^*FS*^. The 95% confidence intervals (CI) of ${{\widehat{R}}_0}^{FS}$ were estimated under the FS assumption by using the binomial distribution for the infected fraction [33,35].

## Results

### Experiments with non-vaccinated calves

Table 1 summarizes the results from the direct and indirect contact experiments with the non-vaccinated calves. FMDV transmission to the contact calves occurred in both experiments.

**Table 1:** **Results of virus isolation, RT-PCR (OPF swabs only), antibody detection and detection of FMD clinical signs**

**Exp** ^**a**^	**Calf ID**	**I:C** ^**b**^	**Group**	**FMDV detection by virus isolation in OPF swabs (in log10 titres),blood(v), urine(u) and faeces(f) samples and by RT-PCR (OPF swabs only, in bold or as ≡)**																				**Antibody detection**		**Clin** ^**c**^ **Inf** ^**d**^	
				**days post infection of the inoculated calves**																				**NS-ELISA**	**VNT**		
				***1***	***2***	***3***	***4***	***5***	***6***	***7***	***8***	***9***	***10***	***11***	***12***	***13***	***14***	***15***	***16***	***17***	***18***	***19***	***20***				
**DC**	3643	I		≡^e^	**2.2** ^f^	**≡**,v^g^	**2.6**,v,u^h^	**3.8**,v,u	**2.0**,u	-	-	-	-	-	-	-	-							+	+	Yes	Yes
	3644	C		**-**	**-**	**-**	-	**1.5**	-,v,u	**2.1**,v	**1.4**,v	≡	**1.7**,u	-	-	**1.2**	≡							+	+	Yes	Yes
	3645	I		**-**	**-**	≡,v	**2.5**,v,f^i^	**2.6**,v	**0.9**,u	**1.2**,u	**0.7**	**0.7**	-	-	-	-	-							+	+	Yes	Yes
	3646	C		**-**	**-**	**-**	-	-	≡	**3.8**	**4.3**,v	**3.0**,v,u	**3.0,u**	-,u	**0.4**	-	-							+	+	Yes	Yes
	3647	I		**-**	≡	**-**	-	-	-	-	-	-	-	-	-	-	-,u							+	+	No	No
	3648	C		**-**	**-**	**-**	-	-	-	-	-	-	-	-	-	-	-							-	-	No	No
	3649	I		**-**	**-**	**0.7**,v	**2.3**,v,u	**3.4**,v	**0.9**,u	-,u	-,u	-	-	-	-	-	-							+	+	Yes	Yes
	3650	C		**-**	**-**	-	-	-	**2.3**,v,u	**1.6**,v,u,f	**0.4**,u,f	≡,u	**0.9**	**0.4**	-,u	-	-							+	+	Yes	Yes
	3651	I		**-**	**-**	**1.4**	≡	**4.2**	**1.7**	**0.4**	-	-	-	-	-	-	-							+	+	Yes	Yes
	3652	C		**-**	**-**	-	-	-	**-**	**-**	0.4	-	-	-	-	-	-							+	+	No	Yes
**IC**	3653	I	A	**-**	**-**	n.t^j^	**1.7**,v,u	**3.4**,v	**2.1**,u															-	-	Yes	Yes
	3654	I	A	**-**	≡	**6.0**,v	**4.6**,v	**3.7**,v,f	**1.7**															-	-	Yes	Yes
	3657	C1	A			-	-	-	-	-	-	-	-	-	-	-	-	-	-	-	-	-	-	-	-	No	No
	3658	C1	A			-	-	-	-	-	-	-	-	-	-	-	-	-	-	-	-	-	-	-	-	No	No
	3661	C2	A						-	-	-	-	-	**3.7**,v	≡,v,u	≡,v,u	**2.4**	≡,u	≡	≡	-	-	-	+	+	Yes	Yes
	3662	C2	A						-	-	-	-	**2.0**	≡	≡	≡,v	**1.3**,v	**3.8**,v,u	**3.5**,v,u	**3.6**,u	≡	-	≡	+	+	Yes	Yes
	3655	I	B	-	-	**2.1**	**5.2**,v	**4.9**,v,u	**2.6**,u															-	-	No	Yes
	3656	I	B	-	0.4	≡,v	**3.2**,v,u	**5.0**,v,u,f	**3.5**,u															-	-	No	Yes
	3659	C1	B			-	-	**1.9**	**2.0**	**3.6**,v,f	**4.9**,v,u	**4.1**,v,u	**3.4**,u	-	**1.2**	-	-	-	-	-	-	-	-	+	+	Yes	Yes
	3660	C1	B			-	-	-	-	0.9	**1.3**,u	**0.7**	**1.0**	-	-	-	-	-	-	-	-	-	-	-	-	Yes	Yes
	3663	C2	B						-	-	-	-	-	-	-	**2.4**	**0.7**	**1.6**	**3.3**,u	**2.3**,u	≡	-	-	+	+	Yes	Yes
	3664	C2	B						-	**0.4**	**1.0**	1.9	**3.0,v**	**5.2**,v	≡,v	**2.6**	**0.4**	≡	-	**-**	-	-	-	+	+	Yes	Yes

^a^Exp=experiment: DC=direct contact, IC=indirect contact; ^b^I=inoculated, C=contact animal; ^c^Clin=clinical signs; ^d^Inf=infectious; ^e^results of virus isolation (VI) and RT-PCR of oral swab sample: - = VI and RT-PCR negative, ≡ = VI negative and RT-PCR positive; ^f^oral swab sample scored positive for FMDV by VI (log10 pfu/mL), RT-PCR positive samples are indicated in **bold**; ^g^v=viraemia: blood sample scored positive for FMDV by VI; ^h^u=urine sample scored positive for FMDV by VI; ^i^f=faeces sample scored positive for FMDV by VI; ^j^n.t.=not tested. Table in which the PCR positive samples are indicated (thus either the ≡ or **VI titres in bold**) in an effort to help with the editorial of this table.

### Direct contact experiment

#### Inoculated calves

FMD clinical signs were observed in 4 of the 5 inoculated calves. Three of these calves (calves 3643, 3645 and 3649) showed fever and had FMD lesions on the tongue. One of these 3 calves (calf 3643) also had hoof lesions, and another (calf 3651) showed FMD lesions on the nose. Three of the clinically infected calves (calves 3643, 3645 and 3649) shed FMDV in OPF, blood and urine (Table 1). One of these 3 calves (calf 3645) also shed FMDV in faeces. The fourth clinically infected calf (calf 3651) shed FMDV in OPF only. All the inoculated calves were positive in OPF by RT-PCR. Antibodies against non-structural proteins and neutralizing antibodies against FMDV were detected in serum samples from all the inoculated calves. Inoculated calf 3647 became subclinically infected, but shed FMDV in urine, was positive for FMDV in OPF by RT-PCR and developed antibodies against non-structural proteins and neutralizing antibodies against FMDV.

#### Contact calves

Clinical signs were observed in the 3 contact calves (calves 3644, 3646 and 3650) that were housed together with inoculated calves 3643, 3645 and 3649. The 3 contact calves showed fever and had FMD lesions on the tongue (calf 3646) and hooves (calves 3644 and 3650); they shed FMDV in OPF, blood and urine (Table 1). One of these 3 calves (calf 3650) also shed FMDV in faeces. Another contact calf (calf 3652) became subclinically infected; it shed FMDV in its OPF. Calves 3644, 3646 and 3650 were positive for FMDV in OPF by RT-PCR. All 4 contact calves in which the virus was detected showed antibodies against non-structural proteins and neutralizing antibodies against FMDV. Calf 3648, in contact with inoculated calf 3647, showed no FMD clinical signs and tested negative for FMDV and for antibodies against FMDV. Thus transmission occurred in 4 of the 5 animal rooms in the direct contact experiment. The only moment infectious virus was recovered from inoculated calf 3647 (from urine) was at 14 dpi, at the day of the end of the experiment. Thus, occurrence of transmission was not possible anymore and this pair of calves (calves 3647 and 3648) was excluded from the estimation of the transmission rate parameters and the reproduction ratio.

### Indirect contact experiment

#### Inoculated calves

Clinical signs were observed in 2 out of 4 inoculated calves (number 3653 and 3654; both in pair IA). These 2 inoculated calves showed fever and 1 of them had lesions on the tongue. The other 2 calves (calves 3655 and 3656; pair IB) showed no FMD specific clinical signs. In all 4 inoculated calves, virus was detected in the OPF (IA and IB). All four secreted and excreted FMDV in their blood, urine and/or faeces (Table 1). They all were positive for FMDV in OPF by RT-PCR. Thus, inoculated calves 3655 and 3656 were subclinically infected. Serum samples from all 4 inoculated calves were obtained only at 0 dpi and 3 dpi; in these samples neither antibodies against non-structural proteins nor neutralizing antibodies against FMDV were detected as expected.

#### Contact calves C1

Contact calves C1 were exposed to the animal rooms that were contaminated by the inoculated calves from 0 to 3 dpi. The contact calves of group C1A (calves 3657 and 3658) did not get infected; no FMD specific clinical signs were seen and both calves tested negative by virus isolation, by RT-PCR and, for antibodies against FMDV. The contact calves of group C1B (calves 3659 and 3660) showed fever and one had FMD lesions on the mouth, tongue, nose and hooves. Both C1B calves had virus detected in their OPF; one of them secreted and excreted virus in blood, urine and faeces, the other one excreted virus in urine. They tested positive for FMDV in OPF by RT-PCR. One C1B calf showed antibodies against non-structural proteins in serum (calf 3660). Both C1B calves showed neutralizing antibodies in serum.

#### Contact calves C2

Contact calves C2 were exposed to the animal rooms that were contaminated by the inoculated calves from 3 to 6 dpi. All the contact calves of groups C2A and C2B showed clinical signs. Three of them showed fever, and all of them showed FMD lesions on the nose and in the mouth. In all 4 calves, virus was detected in their OPF (Table 1); the calves secreted and/or excreted FMDV in the blood (calves 3661, 3662 and 3664) and in urine (calves 3661, 3662 and 3663). They all were positive for FMDV in OPF by RT-PCR. All developed antibodies against non-structural proteins as well as neutralising antibodies. Thus transmission occurred in the indirect contact experiment in 1 of the 2 animal rooms that were contaminated from 0 to 3 dpi and, in both of the animal rooms that were contaminated from 3 to 6 dpi.

### Statistical analysis of virus secretion and excretion

The mean values for the AUC’s, peak of virus shedding and duration of virus shedding (and their ranges) for OPF swabs, urine samples, faeces samples and blood samples for the inoculated group, the direct contact group and the indirect contact groups C1 and C2 are shown in Additional file 3.

No significant difference in log AUC could be determined between the different experimental groups i.e. inoculated, direct contacts, indirect contacts C1 and indirect contacts C2, neither for OPF swabs nor for urine nor for faeces (*p* > 0.05). No significant difference in the maximum FMDV log titres was found between the different experimental groups neither for OPF swabs nor for urine nor for faeces (*p* > 0.05). No significant difference in the duration of FMDV secretion and excretion could be determined between the different experimental groups (*p* > 0.05) (Additional file 3).

### Experiments with vaccinated calves

At day of challenge (0 dpi, 7 days post vaccination), the average virus neutralisation test (VNT) titre against the vaccine strain FMDV Asia-1 Shamir for all the vaccinated calves (including the vaccine controls) was 2.2 ^10^log. The average virus neutralisation test (VNT) titre against the challenge strain FMDV Asia-1 TUR/11/2000 was 1.2 ^10^log.

### Direct contact experiment

After challenge, neither the vaccinated inoculated calves nor the vaccinated contact calves showed clinical signs of FMD and all calves tested negative by virus isolation and RT-PCR. Only 2 inoculated calves (calves 3972 and 3976) developed antibodies against non-structural proteins.

### Indirect contact experiment

After challenge, neither the vaccinated inoculated calves nor the non-vaccinated contact calves showed clinical signs of FMD. All calves tested negative by virus isolation and RT-PCR. Neither the vaccinated inoculated nor the non-vaccinated contact calves showed detectable antibodies against non-structural protein.

### FMDV survival rate (σ)

From the combined published and own experimental data, it was estimated that at 20 °C a 10-fold reduction in FMDV titres occurs in 2.4 days (95% CI: 1.7, 3.3). We calculated the FMDV survival rate (σ) using the lowest (in spiked urine), middle (in spiked faeces) and highest (in spiked buffered solution) estimates obtained at 20 °C. An additional file shows these estimates inside a dashed pointed rectangle (Additional file 4). The estimated time needed for 10-fold reduction in FMDV titres in spiked urine (lowest value) was 0.5 days, indicating an FMDV survival rate (σ) of 0.014 day^−1^. The estimated time needed for 10-fold reduction in FMDV titres in spiked faecal material (middle value) was 2.8 day indicating an FMDV survival rate (σ) of 0.44 day^−1^. The estimated time needed for 10-fold reduction in FMDV titres in spiked buffered solution (highest value) was 8.2 days, indicating an FMDV survival rate (σ) of 0.75 day^−1^. For the quantification of FMDV transmission, we used the middle estimate i.e. σ = 0.44 day^−1^.

### Quantification of FMDV transmission

#### Results of the 1R-SIR model

The transmission rate parameter $\widehat{\beta}$ was 0.67 per day (95% CI: 0.26, 1.8). The average infectious period from the inoculated calves $\widehat{T}$ was 5.5 days (95% CI: 4.5, 6.7). Therefore the estimated reproduction ratio ${{\widehat{R}}_0}^{1\mathrm{R}}$ was 3.7 (95% CI: 1.3, 10.), significantly above 1.

#### Results of the 2R- SIR model

The regression coefficient of f_e_, the extra infectivity contributed by the environment, was not significantly different from 0 which means that *β*_*contact*_ and *β*_*environment*_ are not significantly different. Because *β*_*environment*_/*β*_*contact*_ equalled 1.4 (95% CI 0.14, 14), there is contribution of the environment. Using the most parsimonious model β_*contact*_ and β_*environment*_ were estimated both to be 0.45 per day (95% CI: 0.24, 0.85). Because $\widehat{T}$ was 5.5 days (95% CI: 4.5, 6.7), ${{\widehat{R}}_0}^{2R} contact$ equalled 2.5 (95% CI: 1.3, 5.0). The average infectious period from the contaminated environment $\widehat{\tau}$ was 4.3 days (95% CI: 3.6, 5.2), which leads to a ${{\widehat{R}}_0}^{2R} environment$ of 1.9 (95% CI: 1.007, 3.8). Combination of the two estimates $\left({{\widehat{R}}_0}^{2R} contact+{{\widehat{R}}_0}^{2R} environment\right)$ resulted in ${{\widehat{R}}_0}^{2R}$ equalled to 4.4 (95% CI: 1.5, 7.4), which is significantly above 1. ${\widehat{R}}^{2R}$ was not significantly different from ${{\widehat{R}}_0}^{1R}$ as can be seen from their overlapping confidence intervals. The contribution of the environmental transmission to the total transmission of FMDV was 44% $\left({{\widehat{R}}_0}^{2R} environment/{{\widehat{R}}_0}^{2R}\right)$. The sensitivity analysis, i.e. multiplication of the new infections or cases (C) in E_t_ by either 0 or 0.5, resulted in the same contribution of the environmental transmission (44%). When the lowest and the highest values of σ were used, the contribution of the environmental transmission to the total transmission was estimated to be 31% (when σ = 0.014 day^−1^) and 75% (when σ = 0.75 day^−1^). The sensitivity analysis in which we included a latent period of 1, 2 or 3 days, resulted in higher estimates for β (Additional file 5) and R_0_ (Additional file 6) for the models with a latent period, but the estimated contribution of the environment stayed the same (Additional file 6).

#### Results of the final size model

The ${{\widehat{R}}_0}^{FS}$ equalled 14 (95% CI: 1.3, infinite), which is significantly above 1. Based on the comparison of the confidence intervals, ${{\widehat{R}}_0}^{FS}$ seems to be not significantly different from ${{\widehat{R}}_0}^{1R}$ nor from ${{\widehat{R}}_0}^{2R}$.

#### Experiments with vaccinated calves

After challenge, none of the inoculated or contact calves became infectious; therefore transmission parameters could not be estimated.

## Discussion

In this study, we quantified the contribution of a contaminated environment to the transmission of FMDV and analysed whether vaccination one week prior to inoculation of the calves could block FMDV transmission. We show that using a 2R-SIR model allows FMDV transmission to be quantified in two parts: the direct contact component and the indirect i.e. via the environment component. Our results show that roughly 44% of the transmission of FMDV occurs via the environment, in the days after the calves started secreting and excreting the virus. The contribution of the environment to the transmission of FMDV depends on the FMDV survival rate; if the survival rate is high, the contribution of the environment is higher.

An environment that has previously housed infectious animals can contain FMDV if it is not properly disinfected after the removal of the infectious animals [5] and our study shows that this virus accumulation can cause new infections. As we show, environmental transmission of FMDV plays a role in the total transmission of FMDV also in groups of animals that do have direct contact. Transmission of FMDV has been quantified before in several studies by using a 1R-SIR model [14,18,36-41]. We believe that in all of these studies, transmission occurred through both routes: via direct contact to an infected animal and via indirect contact to a contaminated environment. However within the experimental design of those studies, the role of the environment could not be separated from the role of direct contact on the transmission of FMDV. By using both direct and indirect contact experiments we could employ a 2R-SIR model (that included accumulation of FMDV in the environment) to quantify the contribution of the environment $\left({{\widehat{R}}_0}^{2R}\right)$ to the total transmission of FMDV. As expected, the estimated ${{\widehat{R}}_0}^{1R},\kern0.5em {{\widehat{R}}_0}^{2R}$ and ${{\widehat{R}}_0}^{FS}$ are very similar to each other and moreover, they are similar to the ${\widehat{R}}_0$ (by using a 1R-SIR model) estimated in other direct contact experiments with cattle infected with FMDV O/NET/2001 [18,41]. The consistency of these results indicates that our 2R-SIR model is valid for the estimation of the reproduction ratio and that it is very useful to separate both components i.e. the environment and direct contact transmission, for the quantification of their separate contribution to the transmission of FMDV. Moreover based on the statistical analysis of virus secretion and excretion, the results obtained with the 2R-SIR model are not biased by the route of infection i.e. inoculated and contact infected calves.

In our models, we used an SIR model and we did not incorporate a latent period (then we would have a SEIR i.e. susceptible, exposed, infectious, recovered model), although the data from the virus excretion of the inoculated animals suggest that for this group there is a latent period of approximately 2 days. The main reason why we did not incorporate a latent period in our study is because we did not want to introduce more complexity in the model. Also, incorporation of a latent period affects the estimates for the direct and indirect transmission more or less equally and thus the estimation of the role of the environment (the main interest of this research) was not be affected. Our sensitivity analysis showed that, when a latent period is incorporated in the models, the estimates of the transmission parameters are still “equal” i.e. not significantly different (Additional files 5 and 6). The transmission parameters we provide in Additional files 5 and 6, where a latent period was used, could be useful when the transmission parameters are applied for modelling disease outbreaks and the effect of control measures.

The temporal separation used in our indirect contact experiment allowed us to observe the occurrence of transmission through the environment by taking into consideration virus accumulation in 2 different periods i.e. 0–3 and 3–6 dpi. Temporal separation was also used by Charleston et al. [42] to study FMDV transmission, although they exposed “donor” calves to “recipient” calves by direct contact for 8 hours in separate environments that had been previously disinfected, and thus with no accumulation of virus in the environment. This would, based on our results, reduce transmission of FMDV. They conclude in their study that the occurrence of FMDV transmission is correlated with the presence of clinical signs. However, it has been previously shown that FMDV transmission also can occur before clinical signs are seen [39]. In our study as well, transmission through the environment was caused by one group of calves that contaminated the environment from 0 to 3 dpi but showed no clinical disease. This supports the conclusion that the correlation of FMDV transmission with the presence of clinical signs cannot be generalised to populations, if animals have direct contact to each other for a longer period and/or are present where accumulation of FMDV in the environment is plausible. FMDV transmission may not occur, however, when animals are separated by fences or wooden walls (in pigs [43]; in calves: Charleston et al. (personal communication), [20]), indicating that either exposure to virus secreting and/or excreting animals or exposure to virus contaminated surfaces is important for the occurrence of transmission.

Vaccination can be used as a tool to reduce transmission of FMDV [17]. In our study the calves vaccinated one week prior to inoculation with FMDV did not shed virus. Previously, vaccinating animals 2 weeks prior inoculation with FMDV was reported [18] to reduce FMDV transmission; our results indicate that vaccination reduces FMDV transmission even earlier. As others have demonstrated, vaccination rapidly protects cattle from clinical disease, and reduces virus shedding by infected cattle [44-46]. As our results indicate, vaccination as early as one week before challenge cannot only protect calves against infection but also, can avoid contamination of the environment and so prevent new infections.

In summary, our study shows that the environment is a relevant mechanism in the transmission of FMDV. The quantification of the magnitude of the contribution of transmission via the environment emphasises again that hygiene is an extremely important control measure for FMDV. And that, as already recommended by veterinary authorities, good disinfection of e.g. vehicles, walls and floors previously contaminated by infected animals is necessary to reduce the accumulation of the virus in the environment and therefore FMDV transmission. Also, the data from our experiment give some insight in which secretions and excretions contain FMDV at different times post infection and also this knowledge could be to improve control measures. The accumulation of FMDV in the environment should be taken into account when studying FMDV transmission. Further, the environmental aspect in the transmission of FMDV should be considered during the planning and implementation of measures to control FMD during an outbreak.

## Additional files

Additional file 1:
**On the FMDV survival rate σ.** Detailed calculation of the FMDV survival rate σ, which was calculated using published data on FMDV thermal inactivation combined with own laboratory data [7,20,23-27]. Additional file 2:
**The 2R-SIR model.** Detailed information on the quantification of transmission rate parameters. The transmission rate parameters were calculated using a Generalized Linear Model (GLM) based on an stochastic SIR model. In this additional file we describe the SIR model parameters, the inclusion of an extra route i.e. E to the 1 route SIR-model to calculate the contribution of the environment to the transmission of the infection and, the methodology to quantify the transmission parameters using the GLM model [28,29]. Additional file 3:
**Mean values (plus range) and the Kruskal-Wallis statistics of virus present in secretions, excretions and blood samples, for the inoculated, direct contact and indirect contact groups.** For the Kruskal-Wallis statistics, H is the Kruskal-Wallis test statistic and df the degrees of freedom. Additional file 4:
**Plotted linear regression estimates of the log time (hours) needed for a 10-fold reduction in FMDV titres.** In this additional file we show the obtained times (log hours) that are needed to have a 10-fold reduction in FMDV titres per sample and per temperature. Light blue points correspond to estimates from water; green from buffers; grey from hemal and lymph nodes and bone marrow; black from faeces; red from urine; pink from milk; blue from slurry. Inside the dashed pointed rectangles, only obtained estimates at 20 °C. Red dashed lines, regression lines at 95% CI. Additional file 5:
**Sensitivity analysis considering latent periods.** In this additional file we show results of the estimation of transmission parameters for latent periods of 0 (as used in the paper), 1, 2 and 3 days. Additional file 6:
**Sensitivity analysis considering latent periods.** In this additional file we show results of the estimation of *R*_0_’s for latent periods of 0 (as used in the paper), 1, 2 and 3 days.
