# Supplementary material for: Quantification of transmission of foot-and-mouth disease virus caused by an environment contaminated with secretions and excretions from infected calves
Source: Vet Res. 2015 Apr 17;46(1):43. doi: 10.1186/s13567-015-0156-5 (PMC4404111; doi:10.1186/s13567-015-0156-5)
Supplement: Additional file 1: — On the FMDV survival rate σ. Detailed calculation of the FMDV survival rate σ, which was calculated using published data on FMDV thermal inactivation combined with own laboratory data [7,20,23-27]. [file 13567_2015_156_MOESM1_ESM.docx]

**Additional file 1 On the FMDV survival rate σ**

Published data on FMDV titres of serum samples [[44](#_ENREF_44)], medium [[7](#_ENREF_7),[44](#_ENREF_44)], buffer [[45](#_ENREF_45)], milk [[46](#_ENREF_46),[47](#_ENREF_47)], slurry [[7](#_ENREF_7)], bone marrow [[48](#_ENREF_48)], lymph nodes [[48](#_ENREF_48)] and hemal nodes [[48](#_ENREF_48)] after being exposed to different temperatures, were collected and put in a database.

Further, in a laboratory experiment, we contaminated water, Eagle's Minimum Essential Medium (EMEM), faeces (from calves) and urine (from calves) samples at the starting time (0h) with an inoculum that contained 10 ^6.8^ pfu/mL of FMDV Asia-1 TUR/11/2000 (all in Duplo). All samples were exposed to 4 °C and 20 °C for 0 h, 8 h, 24 h, 48 h, 96 h, 168 h, 336 h, or 504 h. Water and urine, mixed previously with 10% ABII (by volume, v/v), were contaminated with 10% of the FMDV inoculum (v/v). After the required incubation, 800 µL of water or urine was mixed with 200 µL of a 50% FCS, 40% ABII solution and stored at −70 °C until virus isolation analysis. EMEM (containing 10% ABII and 2% FCS) was mixed with 10% ABII (v/v) and contaminated with 10% of the FMDV inoculum (v/v). After the required incubation, samples were stored at −70 °C until virus isolation analysis. Using a sonicator, we mixed and homogenized 50% of faeces with 50% of the FMDV inoculum (w/v). 10% ABII (v/v) was added to the mix. After the required incubation, the faecal mix was suspended 1:10 (by weight, w/v) in EMEM containing 10% FCS, and vortexed with glass beads. After 20 min of incubation at environmental temperature, the suspension was vortexed and centrifuged (3000 × *g* for 15 min). The supernatants were stored at −70 °C until virus isolation analysis. Virus isolation and titration were performed as described before [[20](#_ENREF_20)].

The FMDV titres from serum, medium, buffer, milk, slurry, bone marrow, lymph nodes and hemal nodes exposed to 2.5 °C, 4 °C, 20 °C, 37 °C, 43 °C, 49 °C, 50 °C, 55 °C, 56 °C, 60 °C, 61 °C, 65 °C, 67 °C, 70 °C, 72 °C and/or 80 °C for different intervals of time (from published data), and the obtained FMDV titres from water, medium, faeces and urine exposed to 4 ºC and 20 ºC for different intervals of time (from laboratory data), were recorded. Using the recorded FMDV titres per time of exposure, we calculated how much time is needed to have a 10-fold reduction in FMDV titres by dividing 1 (the logarithmic reduction of interest) by the slope of FMDV titres on time (per sample and per temperature). The obtained times that are needed to have a 10-fold reduction in FMDV titres (in hours) per sample and per temperature, were transformed in logarithmic scale and plotted against temperature (°C) to obtain a regression line (Additional file 3). Then the estimated time that is needed to have a 10-fold reduction in FMDV titres (in days) at 20 °C was used to calculate the FMDV survival rate σ as: 0.1 to the exponential of 1 divided by the necessary days to have a 10-fold reduction in FMDV titres at 20 °C.
