# Supplementary material for: Quantification of transmission of foot-and-mouth disease virus caused by an environment contaminated with secretions and excretions from infected calves
Source: Vet Res. 2015 Apr 17;46(1):43. doi: 10.1186/s13567-015-0156-5 (PMC4404111; doi:10.1186/s13567-015-0156-5)
Supplement: Additional file 2: — The 2R-SIR model. Detailed information on the quantification of transmission rate parameters. The transmission rate parameters were calculated using a Generalized Linear Model (GLM) based on an stochastic SIR model. In this additional file we describe the SIR model parameters, the inclusion of an extra route i.e. E to the 1 route SIR-model to calculate the contribution of the environment to the transmission of the infection and, the methodology to quantify the transmission parameters using the GLM model [28,29]. [file 13567_2015_156_MOESM2_ESM.docx]

**Additional file 2 The 2R-SIR model**

In the 1R-SIR model we use only the data from the direct contact experiment with non-vaccinated calves. The model is: $S\xrightarrow{}I\xrightarrow{}R$

Where susceptible animals (S_t_) are infected with a rate:

$\frac{d(S_{t})}{dt}=-\beta\times\frac{S_{t}\times I_{t}}{N_{t}}$ **Equation 1**

*ß* is the average number of new infections caused by a typical infectious individual per unit of time (day) in a fully susceptible population; *S_t_* is the number of susceptible animals; *I_t_* is the number of infectious animals; and *N_t_* is the total number of animals present at time (t). Division by *N_t_* is done based on the assumption of constant density after comparison of different group sizes [[43](#_ENREF_43)].

Per susceptible animal the number of contacts that lead to infection during a period with the length $\Delta t$, is:

$\beta\times\frac{I_{t}}{N_{t}}\times\Delta t$ **Equation 2**

The 1R-SIR model is analysed as in previously reported studies [[23](#_ENREF_23),[24](#_ENREF_24)].

In the 2R-SIR model we use data from both the direct contact experiment and the indirect contact experiment. In this model we included an extra route to the 1R-SIR model: E. The model is: $S\xrightarrow{}I\xrightarrow{}R$

$$\begin{matrix} \downarrow\\ E \end{matrix}$$

In this case calves are exposed to both infectious animals (I_t_) and/or to virus coming from infectious animals via the environment (E_t_) (see Figure 2 in manuscript). E_t_ is based on the secretion and excretion of FMDV by the infectious animals on previous days as well as on the remaining virus in the environment. We therefore include the FMDV survival rate (σ), described in Additional file 2, to correct for the decrease of FMDV in time, thus:

**Equation 3**

Thus the rate of infection per susceptible individual during a period with the length becomes:

*ß_contact+environment_* $\times\frac{I_{t}+E_{t}}{N_{t}}\times\Delta t$ **Equation 4**

*ß_contact+environment_* is a combined transmission rate parameter for contact exposure to an infected animal and for contact exposure to a contaminated environment (for its calculation we used data from both direct contact and indirect contact experiments).

By replacing *ß_contact+environment_* by $e^{c_{0}+f_{e}\times c_{1}}$this can be rewritten as:

$e^{c_{0}+f_{e}\times c_{1}}\times\frac{I_{t}+E_{t}}{N_{t}}\times\Delta t$ **Equation 5**

Where *f_e_* is the fraction of transmission by the environment and its regression coefficient measures the extra infectivity contributed by the environment. If the contribution of the environment is zero, then *f_e_* becomes zero (because *E_t_* is zero) and *ß_contact+environment_* = *ß_contact_* equal to $e^{c_{0}}$. If there are no infectious animals present, then *f_e_* is 1 and *ß_contact+environment_* = *ß_environmen_*_t_ equal to $e^{c_{0}+c_{1}}$.

The probability that a single susceptible animal becomes infected is then binomial distributed with:

$p=1-e^{-e^{c_{0}+f_{e}\times c_{1}}\times\frac{I_{t}+E_{t}}{N_{t}}\times\Delta t}$ **Equation 6**

The data are analysed with a Generalised Linear Model (GLM) with a complementary log-log link, thus we take the log(-log(1-p)). The expected value of C/S when applying the link function is:

$c_{0}+f_{e}\times c_{1}+\log(\frac{I_{t}+E_{t}}{N_{t}}\times\Delta t)$ **Equation 7**

So this is a GLM with offset:

$\log(\frac{I_{t}+E_{t}}{N_{t}}\times\Delta t)$ **Equation 8**

With S as binomial total, a binomial error function, and with explanatory variable *f_e_* (the infectivity contributed by the environment): $\frac{E_{t}}{I_{t}+E_{t}}$.
