# Supplementary material for: Quantification of transmission of foot-and-mouth disease virus caused by an environment contaminated with secretions and excretions from infected calves
Source: Vet Res. 2015 Apr 17;46(1):43. doi: 10.1186/s13567-015-0156-5 (PMC4404111; doi:10.1186/s13567-015-0156-5)
Supplement: Additional file 3: — Mean values (plus range) and the Kruskal-Wallis statistics of virus present in secretions, excretions and blood samples, for the inoculated, direct contact and indirect contact groups. For the Kruskal-Wallis statistics, H is the Kruskal-Wallis test statistic and df the degrees of freedom. [file 13567_2015_156_MOESM3_ESM.docx]

**Additional file 3 Mean values (plus range) and the Kruskal-Wallis statistics of virus present in secretions, excretions and blood samples, for the inoculated, direct contact and indirect contact groups.** H is the Kruskal-Wallis test statistic and df the degrees of freedom.

|  | **Mean values (range)** | | | |  | **Kruskal-Wallis statistics** | | |
| --- | --- | --- | --- | --- | --- | --- | --- | --- |
| **Measured variables** | **Inoculated group** | **Direct contact group** | **Indirect contact groups** | |  | **H** | **df** | ***P*-value** |
|  |  |  | **C1** | **C2** |  |  |  |  |
| ***AUC* *(log_10_ titres)*** |  |  |  |  |  |  |  |  |
| OPF swabs | 8.8 (0, 15) | 5.7 (0, 15) | 6.2 (0, 21) | 6.1 (2.6, 14) |  | 2.32 | 3 | 0.51 |
| Urine | 2.3 (0, 5.1) | 2.1 (0, 6.2) | 1.8 (0, 5.3) | 1.0 (0, 4.0) |  | 1.60 | 3 | 0.66 |
| Faeces | 0.3 (0, 1.2) | 0.4 (0, 2.1) | 0.2 (0, 0.2) | 0 |  | 1.54 | 3 | 0.67 |
| Blood | 5.8 (0,9.5) | 4.0 (0, 8.7) | 2.1 (0, 8.3) | 4.6 (0, 9.8) |  | 2.35 | 3 | 0.50 |
| ***Maximum FMDV excretion (log_10_ titres)*** |  |  |  |  |  |  |  |  |
| OPF swabs | 3.7 (0, 6.0) | 1.8 (0, 4.3) | 1.6 (0, 4.9) | 4.0 (3.3, 5.2) |  | 5.53 | 3 | 0.14 |
| Urine | 1.2 (0, 2.6) | 0.7 (0, 1.9) | 1.1 (0, 2.7) | 1.7 (0, 2.5) |  | 1.81 | 3 | 0.61 |
| Faeces | 0.3 (0, 1.2) | 0.3 (0, 1.7) | 0.2 (0, 0.9) | 0 |  | 1.54 | 3 | 0.67 |
| Blood | 2.5 (0, 3.6) | 1.9 (0, 3.3) | 0.9 (0, 3.7) | 2.2 (0, 3.4) |  | 1.65 | 3 | 0.65 |
| ***Duration of FMDV excretion (days)*** |  |  |  |  |  |  |  |  |
| OPF swabs | 4.0 (0, 6) | 4.4 (0, 9) | 3.0 (0, 8) | 6.3 (4, 8) |  | 3.01 | 3 | 0.39 |
| Urine | 2.1 (0, 5) | 3.0 (0, 7) | 1.0 (0, 3) | 2.3 (0, 4) |  | 1.68 | 3 | 0.64 |
| Faeces | 0.3 (0, 1) | 0.4 (0, 2) | 0.3 (0, 1) | 0 |  | 1.52 | 3 | 0.68 |
| Blood | 2.1 (0, 3) | 1.4 (0, 3) | 0.8 (0, 3) | 2.5 (0, 4) |  | 4.03 | 3 | 0.26 |
