# Supplementary figures and images for: Quantification of transmission of foot-and-mouth disease virus caused by an environment contaminated with secretions and excretions from infected calves
Source: Vet Res. 2015 Apr 17;46(1):43. doi: 10.1186/s13567-015-0156-5 (PMC4404111; doi:10.1186/s13567-015-0156-5)

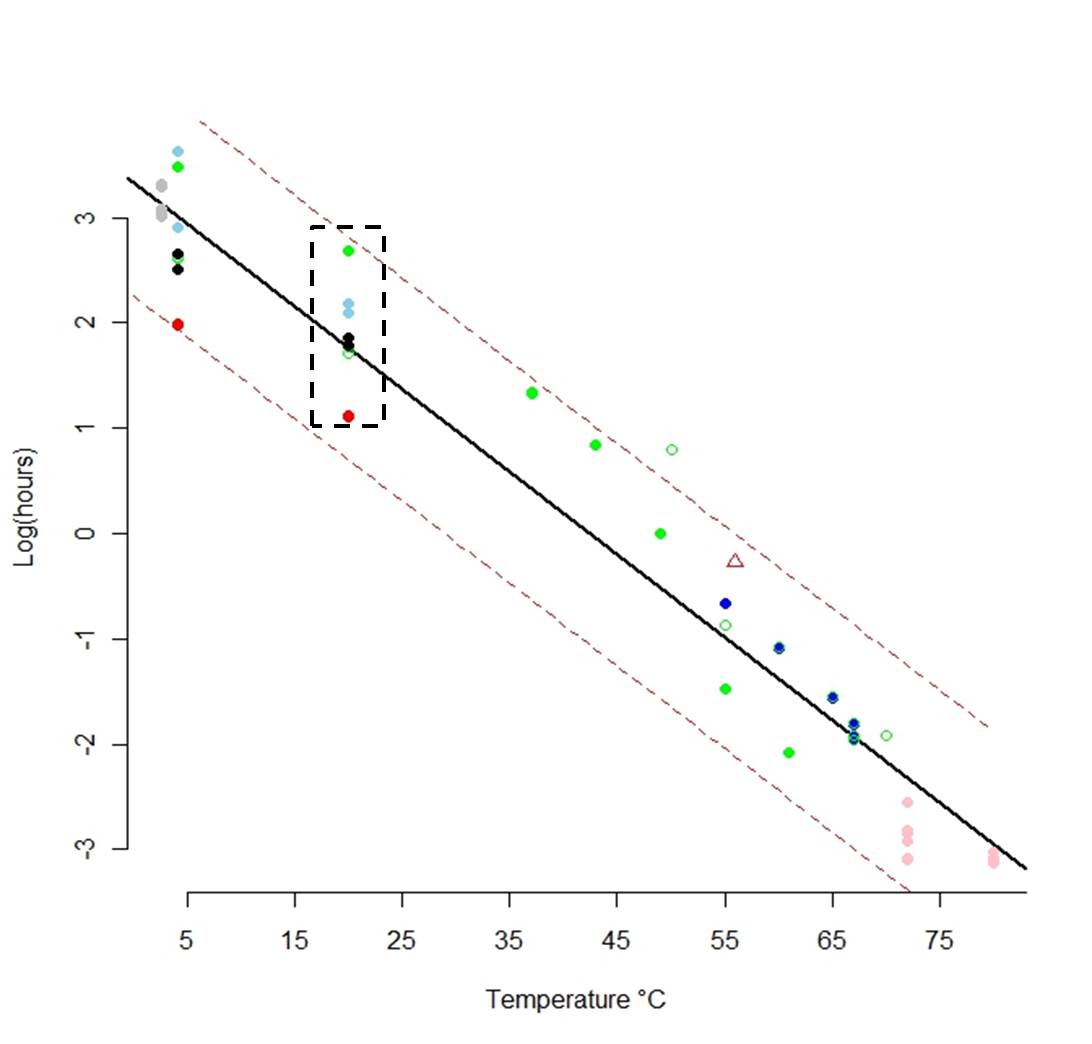

Supplement: Additional file 4: — Plotted linear regression estimates of the log time (hours) needed for a 10-fold reduction in FMDV titres. In this additional file we show the obtained times (log hours) that are needed to have a 10-fold reduction in FMDV titres per sample and per temperature. Light blue points correspond to estimates from water; green from buffers; grey from hemal and lymph nodes and bone marrow; black from faeces; red from urine; pink from milk; blue from slurry. Inside the dashed pointed rectangles, only obtained estimates at 20 °C. Red dashed lines, regression lines at 95% CI. [file 13567_2015_156_MOESM4_ESM.jpeg]
