# Supplementary material for: Quantification of transmission of foot-and-mouth disease virus caused by an environment contaminated with secretions and excretions from infected calves
Source: Vet Res. 2015 Apr 17;46(1):43. doi: 10.1186/s13567-015-0156-5 (PMC4404111; doi:10.1186/s13567-015-0156-5)
Supplement: Additional file 5: — Sensitivity analysis considering latent periods. In this additional file we show results of the estimation of transmission parameters for latent periods of 0 (as used in the paper), 1, 2 and 3 days. [file 13567_2015_156_MOESM5_ESM.docx]

**Additional file 5 Sensitivity analysis considering latent periods.**

| Latent period (days) | *β_contact_* | *β_environment_* | Ratio^1^ | *β_overall_^2^* | N^3^ | Number of contact infections for each number of latent days | | | |
| --- | --- | --- | --- | --- | --- | --- | --- | --- | --- |
|  |  |  |  |  |  | 0 | 1 | 2 | 3 |
| 0 | 0.373 | 0.523 | 1.404 | 0.453 | 47 | 10 |  |  |  |
| 1 | 0.459 | 0.753 | 1.640 | 0.625 | 40 | 1 | 9 |  |  |
| 2 | 0.868 | 0.928 | 1.071 | 0.909 | 33 | 1 | 1 | 8 |  |
| 3 | 1.280 | 1.258 | 0.983 | 1.265 | 29 | 1 | 1 | 3 | 5 |

^1^ Ratio = *ß_environment_/ß_contact_*

^2^ This is the beta estimate for contact and environment when the two are not significantly

different which is the case here for all latent periods.

^3^ N = number of rows in the dataset used in the GLM analysis
