# Supplementary material for: Quantification of transmission of foot-and-mouth disease virus caused by an environment contaminated with secretions and excretions from infected calves
Source: Vet Res. 2015 Apr 17;46(1):43. doi: 10.1186/s13567-015-0156-5 (PMC4404111; doi:10.1186/s13567-015-0156-5)
Supplement: Additional file 6: — Sensitivity analysis considering latent periods. In this additional file we show results of the estimation of R 0’s for latent periods of 0 (as used in the paper), 1, 2 and 3 days. [file 13567_2015_156_MOESM6_ESM.docx]

**Additional file 6 Sensitivity analysis considering latent periods.**

| Latent period (days) | $\hat{R}$*_0_^1R^* | $\hat{R}$*_0_^2R^_contact_* | $\hat{R}$*_0_^2R^_environment_* | $\hat{R}$*_0_^2R^* | Environmental contribution to transmission (%) |
| --- | --- | --- | --- | --- | --- |
| 0 | 3.7 <1.3, 10> | 2.5 <1.3, 4.8> | 1.9 <1.0, 3.8> | 4.4 <1.5, 7.4> | 44 |
| 1 | 5.6 <2.0, 15> | 3.4 <1.8, 6.7> | 2.7 <1.4, 5.2> | 6.1 <3.2, 9.1> | 44 |
| 2 | 12 <4.2, 34> | 5.0 <2.5, 9.8> | 3.9 <2.0, 7.7> | 8.9 <5.9, 12> | 44 |
| 3 | 17 <5.8, 53> | 6.9 <3.5, 14> | 5.4 <2.8, 11> | 5.4 <2.8, 11> | 44 |
